# Interdisciplinarity in Cardiovascular Diseases—A Necessity Alongside the Heart Team

**DOI:** 10.3390/life15020193

**Published:** 2025-01-28

**Authors:** Mariana Floria, Diana-Elena Floria, Anca Victorița Trifan, Daniela Maria Tănase

**Affiliations:** 1 Department of Internal Medicine, Grigore T. Popa University of Medicine and Pharmacy, 700111 Iasi, Romania; floria.mariana@umfiasi.ro (M.F.); anca.trifan@umfiasi.ro (A.V.T.); daniela.tanase@umfiasi.ro (D.M.T.); 2 Clinic of Internal Medicine, St. Spiridon Emergency Hospital Iasi, 700111 Iasi, Romania; 3 Institute of Gastroenterology and Hepatology, St. Spiridon Emergency Hospital Iasi, 700111 Iasi, Romania

Cardiovascular disease often requires multidisciplinary collaboration in order to achieve an appropriate diagnosis and optimal patient outcomes. As pathophysiological mechanisms such as atherosclerosis of micro- or macro-vasculature and autoimmunity are present in many cardiovascular diseases, these can progress to systemic diseases by affecting multiple organs and systems. This further highlights the importance of recognizing syndromes within the diagnosis of systemic diseases and highlights the essential role of interdisciplinarity in managing cardiovascular conditions. Diseases such as recurrent pericarditis, COVID-19-associated cardiovascular complications, coronary artery disease, or congenital heart disease still inspire extensive preclinical and clinical research. These studies continue to pave the way for novel therapeutic strategies and diagnostic advancements.

Recurrent pericarditis markedly impacts patients’ quality of life, given the need for repeated emergency department visits and hospitalizations related to disease evolution or treatment complications. Therapeutic options are still rather limited; the mechanisms of action, treatment protocols, safety, and efficacy of IL-1 blockers still comprise controversial topics. IL-1 blockers may represent a paradigm shift in terms of cost/benefit ratio in patients with multiple recurrences of pericarditis [1]. Despite the recommendations of the latest clinical guidelines on pericardial syndromes [2], the full dose administration protocol is not universally accepted, nor is there a progressive dose reduction protocol. The narrative review by Lazarou E. et al. [1] offers a comprehensive synthesis of the progress made in understanding recurrent pericarditis, particularly focusing on the role of IL-1-driven chronic inflammation.

Chronic inflammation and endothelial dysfunction are closely linked. Given the strong association between vascular dysfunction and ischemia, coronary artery disease can be accompanied by retinal changes. The systematic review and meta-analysis by Rusu A.C. et al. [3] highlighted the fact that the most important retinal alterations compared to healthy controls are reduced macular superficial and vascular density, retinal layer thinning, and increased foveal avascular zone area. These changes are linked to vascular changes, including the central retinal artery and choriocapillaris. Endothelial dysfunction was recognized as a key factor in both systemic atherosclerosis and age-related macular degeneration. Both retinal and coronary microvasculature are similarly impacted by cardiovascular risk factors such as hypertension, diabetes, dyslipidemia, and obesity, leading to endothelial disruption, reduced nitric oxide production, and impaired angiogenesis. Retinal micro-vascularization is strongly influenced by cardiovascular risk factors and severity of coronary artery disease. In patients with acute coronary syndromes, significant associations have been observed between retinal vessel density and cardiovascular risk profiles, as well as left ventricular ejection fractions [4]. Specific retinal microvascular features, such as fractal dimension and vascular density, can indicate an elevated risk of coronary artery diseases, even in individuals without a prior documented diagnosis. Thus, retinal microvascular changes could potentially serve as diagnostic and predictive tools for cardiovascular disease. Superficial capillary plexus vessel density in the temporal and nasal perifovea and deep capillary plexus density in the inferior parafovea may be used as such predictive markers. Advanced imaging techniques, such as optical coherence tomography (OCT) and optical coherence tomography angiography (OCTA), are highly effective in screening and evaluating angiopathy severity. These methods can be automated and integrated with clinical risk factors and coronary evaluations, providing useful diagnostic insights. Their implementation could be particularly useful in conditions like Ischemia with No Obstructive Arteries (INOCAs), characterized by microvascular dysfunction in the absence of coronary blockages. However, ongoing debate persists regarding whether retinal microvascular anomalies precede coronary lesions or occur concurrently.

Vascular involvement in the context of COVID-19 infection encompasses a wide range of manifestations, from endothelial injury to vascular damage and coagulation disorders [4]. The primary entry point for the SARS-CoV-2 virus is through the angiotensin-converting enzyme 2 receptor, which is expressed not only in the respiratory tract, but also in endothelial cells throughout the body. The infection triggers a cascade of events, including endothelial dysfunction, inflammation, hypercoagulability, and immune system alterations. These effects can occur both during the acute phase of the infection and in the long term, being more pronounced in specific organs or with systemic consequences. Over time, this endothelial dysfunction has the potential to impact the entire cardiovascular system in certain patients, with potential severe micro- and macrovascular consequences. The review published by Karakasis P. et al. [5] underlined the complex relationship between the SARS-CoV-2 viral infection and vascular damage. An overreactive acute response or prolonged inflammation may induce cardiovascular complications. The occurrence of acute endothelitis, followed by hypersensitive leukocytoclastic vasculitis, can amplify antibody and autoantibody production, exacerbating a preexisting hypercoagulable state similar to antiphospholipid syndrome. This progression highlights a potential pathway for the transition from inflammation to hypercoagulation in COVID-19 disease. COVID-19 patients with extensive coronary calcification scores may have worse prognoses, while those with aortic aneurysms may experience an accelerated rate of progression. Therapeutic strategies include anticoagulants (primarily low-molecular-weight heparins), anti-inflammatory agents (such as steroids, IL-6 inhibitors, interferon-α2b, and JAK inhibitors), antiviral agents (including ribonucleic acid-dependent RNA polymerase inhibitors), monoclonal antibodies, kinase inhibitors, intravenous immunoglobulin, and convalescent plasma [5]. Some innovative therapies are currently being assessed in ongoing clinical trials. Currently, vaccines have been incorporated into the arsenal of preventive measures. These include viral vector-based vaccines, inactivated or recombinant protein-based vaccines, nucleic acid-based vaccines, and virus-like particles [5]. COVID-19 infection involves a wide range of pathophysiological changes, rendering it rather difficult to categorize into strictly short- or long-term effects. Proper treatment strategies, anticoagulation therapy, and vaccinations are essential steps in improving patient outcome. In this context, prompt detection and management of high-risk individuals with vascular complications is mandatory.

The relationship between cardiovascular disease and inflammation was also investigated by Gosav E.M. et al. [6], highlighting the role of the neutrophil-to-lymphocyte ratio as a potential biomarker for predicting and prognosticating cardiovascular disease outcomes. White blood cells (neutrophil, lymphocyte, and monocyte counts) are independent risk factors for mortality, and the neutrophil-to-lymphocyte ratio is a significant predictor of outcomes. The neutrophil-to-lymphocyte ratio combines two essential aspects of the immune function: the number of neutrophils reflect inflammation, while lymphocyte counts indicate adaptive immunity. The neutrophil-to-lymphocyte ratio is a readily available and inexpensive tool that can be easily calculated, serving as a prognostic and predictive biomarker in clinical practice. The review by Gosav E.M. et al. [6] provides insight into the latest data regarding prognostic and predictive roles of the neutrophil-to-lymphocyte ratio in various cardiovascular outcomes. It also highlights certain gaps in the literature, encouraging more research in order to fully understand the utility of the neutrophil-to-lymphocyte ratio in cardiovascular disease management.

Over 80% of deaths arising in the context of cerebrovascular disease are caused by cardiac events and strokes, with a third of these deaths occurring in young adults [7]. These conditions are the leading cause of mortality worldwide, responsible for around 17.9 million deaths annually; this represents 32% of all deaths, especially in developed and middle-income countries [7]. Disorders such as coronary heart disease, cerebrovascular or peripheral vascular disease markedly impact the quality of life of affected individuals and their families. The significant morbidity and mortality associated with cerebrovascular diseases comprise a major global health concern, posing substantial socio-economic challenges. According to Rodrigues F. et al. [8], certain countries experienced substantial economic and social shifts, leading to notable disparities within their population. These changes amplified the challenges associated with cerebrovascular and cardiovascular diseases, underscoring the need for tailored interventions and strategies to mitigate these escalating health concerns. While established risk factors include obesity, diabetes, hypertension, alcohol consumption, and smoking, other factors such as physical inactivity, suboptimal educational levels, health literacy, and dietary inadequacies pose greater challenges for assessment and quantification. Given the particularities of each country, cohort studies that investigate these factors and national prevention programs are essential.

Harmful alcohol consumption represents a modifiable risk factor for cardiovascular disease. While moderate red wine consumption (one to two drinks per day, maximum 3 times per week) may have cardiovascular benefits, such as increasing high-density lipoprotein cholesterol levels and possessing anti-inflammatory properties, these benefits can be overshadowed by the consequences of excessive use. Chronic excessive alcohol intake can promote the development of a range of cardiovascular issues, including but not limited to hypertension, atrial fibrillation, and heart failure. Heavy or episodic drinking was associated with conditions such as hemorrhagic stroke, atrial fibrillation, and cardiomyopathy [9]. The underlying mechanisms driving these effects involve the direct toxic impact on cardiac myocytes, disruptions in blood pressure regulation, and the facilitation of inflammation and oxidative stress. The relationship between excessive alcohol use and cardiovascular disease is further enhanced by the dose–response nature of this association. This suggests that even moderate drinking could pose health risks in certain individuals or in the context of preexisting conditions. Understanding these individual variances, particularly as they relate to age and other risk factors, is essential in tailoring effective prevention and treatment strategies. In younger individuals, risky behaviors associated with higher alcohol consumption can lead to acute incidents such as accidents and injuries, in addition to potential long-term health issues. In contrast, older adults may face accelerated health declines due to age-related changes in liver function that hinder alcohol metabolism, making them especially vulnerable to the detrimental effects of alcohol on cardiovascular health. According to Georgescu O.S. et al. [10], the impact of alcohol consumption depends on factors such as drinking patterns, age, gender, and individual health conditions. The observed J-shaped relationship between alcohol consumption and cardiovascular conditions highlights the complexity of these effects, hence the need for a nuanced perspective [9]. Public health guidelines should emphasize moderation and, when appropriate, advocate for abstinence, particularly among higher risk populations. A holistic approach to lifestyle modifications, targeting both alcohol consumption and other modifiable risk factors, is essential in improving cardiovascular outcomes and promoting overall health. During their comprehensive evaluation of patients, healthcare providers should incorporate an assessment of an individual’s alcohol consumption patterns, alongside other lifestyle factors [10]. Interventions aimed at reducing alcohol intake could significantly lower the overall cardiovascular risk in both younger and older populations. In a recently published systematic review, a J-shaped relationship was observed between alcohol consumption and arterial stiffness [9].

Increased arterial stiffness is a marker of cardiovascular risk. Carotid–femoral pulse wave velocity is the gold standard for assessing arterial stiffness [11]. Higher arterial stiffness levels are associated with an increased risk of cardiovascular events in patients with chronic kidney disease and those undergoing dialysis [12]. Monitoring the carotid–femoral pulse wave velocity in peritoneal dialysis patients may be useful in risk assessment and tailored interventions, given their heightened susceptibility to cardiovascular disease [12]. Understanding vascular health can enable timely interventions such as lifestyle modifications and adjustments to medication regime or dialysis protocols. Additionally, addressing vascular calcifications related to mineral and bone disorders is important; interventions targeting calcium and phosphate balance, alongside treatments such as vitamin K2 and statins, may reduce calcifications and improve patient outcomes. Regular periodic assessment of arterial stiffness and calcifications is essential in enhancing cardiovascular health and reducing cardiovascular disease-related morbidity and mortality [12]. Moreover, malondialdehyde-oxidized LDL might serve as an indicator of oxidative stress and inflammation, rendering it an important biomarker for assessing cardiovascular risk, particularly in individuals with chronic conditions such as kidney disease, diabetes, and obesity [11,12]. It is thought to be a promoting factor for atherogenesis by enhancing oxidized LDL uptake by macrophages, leading to foam cell formation and arterial plaque development, and by inducing endothelial dysfunction. High levels of malondialdehyde-oxidized LDL can help identify individuals at increased risk for cardiovascular events, which may prompt timely intervention [12]. Overall, according to Chang Y.-C et al. [12], measuring malondialdehyde-oxidized LDL serum levels can provide valuable insights into cardiovascular risk and guide preventive strategies.

The autonomic nervous system plays an essential role in regulating cardiovascular responses. During pregnancy, significant physiological changes occur, including increases in blood volume, cardiac output, and heart rate, in order to meet the metabolic demands of both the mother and fetus. These changes are achieved through vasodilation with decreased systemic vascular resistance, influenced by hormones like progesterone and relaxin. Closely monitoring the balance between sympathetic and parasympathetic activation is a key factor in preventing complications such as gestational hypertension and preeclampsia, according to the research of Bogdan A. et al. [13]. The authors found a rather high prevalence of autonomic and cardiac dysfunctions within this population, which could have serious implications for both cardiovascular health and clinical management. Heart rate variability serves as a useful metric in evaluating autonomic nervous system regulation of the cardiovascular system [14]. It has been widely utilized as a prognostic tool in various cardiac conditions and it is instrumental in understanding how autonomic imbalances may influence other health conditions as well [14]. The study conducted by Bogdan A. et al. [13] focused on the relationship between heart rate variability, cardiac performance, and hypertensive disorders of pregnancy. The authors highlighted that heart rate variability and global longitudinal strain are effective non-invasive methods for identifying autonomic dysfunction and cardiac performance impairments in pregnant women affected by hypertensive disorders. Autonomic nervous system regulation appears to be significantly affected in pregnancies complicated by hypertension, emphasizing the need for careful monitoring [13]. Therefore, incorporation of heart rate variability and global longitudinal strain assessments into routine evaluations of patients with hypertensive disorders of pregnancy could aid proper evaluation of their cardiovascular health and inform appropriate clinical management. Overall, understanding the complex cardiovascular adaptations during pregnancy is essential in ensuring the optimal outcomes of both the mother and child.

A retrospective study by Condello I. et al. [15] highlights the promising role of phosphorylcholine-coated circuits in cardio-pulmonary bypass during cardiac surgery. By providing a biocompatible coating [16], phosphorylcholine may help mitigate some of the complications traditionally associated with cardiopulmonary bypass, such as thrombotic events and systemic inflammation. The use of phosphorylcholine-coated circuits demonstrates favorable changes in laboratory parameters, with comparable safety outcomes compared to non-coated circuits. In this study, no thrombotic complications or circuit-related adverse events were observed. Efficiency metrics—such as blood loss, clotting, and the integrity of cardio-pulmonary bypass circuits—were consistent across both the phosphorylcholine-coated and non-coated groups. This suggests that coating does not impair the performance of the cardio-pulmonary bypass system. These findings suggest that using phosphorylcholine-coated circuits could potentially decrease reliance on costly blood products and resources often used in complex surgical cases [15]. While the initial results are encouraging, the study underscores the necessity for further research to explore the broader implications of phosphorylcholine on patient outcomes and the overall performance of cardio-pulmonary bypass circuits. Incorporation of phosphorylcholine in cardio-pulmonary bypass circuits could represent a significant development in surgical practices by enhancing biocompatibility and minimizing thrombotic risks, thereby improving patient outcomes. Further research is encouraged to substantiate these findings and refine the use of phosphorylcholine in clinical settings.

Congenital heart diseases rank among the most prevalent congenital anomalies, affecting approximately 1% of all live-born infants [17]. The etiology of congenital heart diseases is complex, involving both genetic and environmental factors. Environmental teratogens, including certain medications or viral infections, and maternal conditions like obesity and diabetes, can contribute to the development of congenital heart disease. Genetic factors also play a significant role, as evidenced by the increased recurrence risk within families and the established associations between congenital heart disease and chromosomal abnormalities. However, despite advancements in research, the underlying cause of congenital heart disease remains unknown in approximately 60% of cases [17]. To enhance diagnostic accuracy, current guidelines advocate for the use of chromosomal microarray analysis and next-generation sequencing to identify potential genetic causes of congenital heart disease [17]. Although current diagnostic methods (including cytogenetic techniques such as karyotyping and copy number variant analysis) are useful, they yield a genetic diagnosis in only 18–36% of patients with congenital heart diseases [15]. The success rate of genetic testing varies depending on factors such as specific type of congenital heart defect, presence of associated extra-cardiac anomalies, and the technology employed for testing. Peterlin A. et al. [18] assessed the diagnostic performance of chromosomal microarray analysis and next-generation sequencing in a cohort of neonates with both isolated and syndromic congenital heart disease spanning over 10 years. The authors emphasize the need for comprehensive genetic analyses to better understand the molecular genetic pathology associated with congenital heart disease. Systematic adoption of advanced genetic testing techniques (such as whole-genome sequencing, optical genome mapping, and long-read sequencing) holds promise for enhancing diagnostic yields [18]. A timely genetic diagnosis is crucial for identifying syndrome-related comorbidities, guiding prognosis, evaluating reproductive genetic risks, and facilitating genetic testing for family members.

In conclusion, a key mechanism in cardiovascular diseases is represented by acute or chronic inflammation. Systemic inflammation not only impacts the cardiovascular system but also promotes thrombosis, leading to consequences beyond the heart. When addressing risk factors that promote atherosclerosis in micro- or macro-circulation, both in the short and long term, the impact on other territories beyond the heart is inevitable. Therefore, understanding pathophysiological mechanisms and systemic consequences is essential. Interdisciplinary collaboration has become crucial in cardiovascular disease management, complementing the efforts of the heart team.

## Data Availability

Not applicable.
